# Prenatal Iodine Intake and Maternal Pregnancy and Postpartum Depressive and Anhedonia Symptoms: Findings from a Multiethnic US Cohort

**DOI:** 10.3390/nu16111771

**Published:** 2024-06-05

**Authors:** Aderonke A. Akinkugbe, Yueh-Hsiu Mathilda Chiu, Srimathi Kannan, Veerle Bergink, Rosalind J. Wright

**Affiliations:** 1 Department of Environmental Medicine and Climate Science, Icahn School of Medicine at Mount Sinai, New York, NY 10029, USA; 2 Institute for Climate Change, Environmental Health and Exposomics, Icahn School of Medicine at Mount Sinai, New York, NY 10029, USA; 3 Division of Metabolism, Endocrinology, and Diabetes, Department of Internal Medicine, University of Michigan, Ann Arbor, MI 48109, USA; 4 Department of Psychiatry, Icahn School of Medicine at Mount Sinai, New York, NY 10029, USA; 5 Department of Public Health, Icahn School of Medicine at Mount Sinai, New York, NY 10029, USA

**Keywords:** iodine intake, pregnancy, postpartum, depressive symptoms, anhedonia, pregnancy cohort

## Abstract

**Objective**: Emerging evidence suggests that essential trace elements, including iodine, play a vital role in depressive disorders. This study investigated whether prenatal dietary iodine intake alone and in combination with supplemental iodine intake during pregnancy were associated with antepartum and postpartum depressive and anhedonia symptoms. **Methods**: The study population included 837 mothers in the PRogramming of Intergenerational Stress Mechanisms (PRISM) study. The modified BLOCK food frequency questionnaire was used to estimate prenatal dietary and supplemental iodine intake, while the 10-item Edinburg Postpartum Depression Scale (EPDS) ascertained depressive symptoms. Analyses considered the global EPDS score and the anhedonia and depressive symptom subscale scores using dichotomized cutoffs. Logistic regression estimating odds ratios and 95% confidence intervals (CIs) assessed associations of iodine intake in the second trimester of pregnancy and 6-month postpartum depressive and anhedonia symptoms considering dietary intake alone and combined dietary and supplementary intake in separate models. **Results**: Most women were Black/Hispanic Black (43%) and non-Black Hispanics (35%), with 39% reporting a high school education or less. The median (interquartile range, IQR) dietary and supplemental iodine intake among Black/Hispanic Black (198 (115, 337) µg/day) and non-Black Hispanic women (195 (126, 323) µg/day) was higher than the overall median intake level of 187 (116, 315) µg/day. Relative to the Institute of Medicine recommended iodine intake level of 160–220 µg/day, women with intake levels < 100 µg/day, 100–<160 µg/day, >220–<400 µg/day and ≥400 µg/day had increased adjusted odds of 6-month postpartum anhedonia symptoms (aOR = 1.74 (95% CI: 1.08, 2.79), 1.25 (95% CI: 0.80, 1.99), 1.31 (95% CI: 0.82, 2.10), and 1.47 (95% CI: 0.86, 2.51), respectively). The corresponding estimates for postpartum global depressive symptoms were similar but of smaller magnitude. **Conclusions:** Prenatal iodine intake, whether below or above the recommended levels for pregnant women, was most strongly associated with greater anhedonia symptoms, particularly in the 6-month postpartum period. Further studies are warranted to corroborate these findings, as dietary and supplemental iodine intake are amenable to intervention.

## 1. Introduction

Mood disorders are common in the perinatal period. Perinatal maternal depression includes major and minor episodes during pregnancy (i.e., antenatal) and/or within the first 12 months after delivery (i.e., postpartum). The prevalence of antenatal depression is approximately 20%, while the prevalence of postpartum depression (PPD) ranges from 12 to 18% [1]. A recent report showed that the pooled global prevalence of PPD was 17.7%. PPD subtypes include an estimated 12-month prevalence of 9% for unipolar major depressive disorder and 3% for bipolar disorder [2].

Recent evidence underscores the need to consider the anhedonia depressive subtype, indexed as reduced interest or pleasure to stimuli previously perceived as rewarding consequent to impairment of the effort-based reward system [3]. Anhedonia has been reported in approximately 30% and 50% of individuals with unipolar and bipolar depressive disorder, respectively [4]. Mechanisms underlying anhedonia are distinct from depression. Anhedonia, which may be a surrogate indicator of future depressive illness, has been associated with increased depressive symptom severity and is a negative predictor of treatment response [2]. Studies on anhedonia in the postpartum period remain sparse, but it is estimated to occur in 23% of mothers [5,6]. In addition, women with symptoms suggestive of an anhedonia subtype in pregnancy are more likely to report depression during the postpartum period [2].

Mood disorders in the peripartum period are associated with a significant impact on maternal morbidity and mortality [7,8]. Psychological dysfunction also has implications for the offspring of affected women, being associated with suboptimal interactions between mothers and infants with consequent adverse effects on child cognitive, social, and emotional development, including high levels of child internalizing behaviors [9]. Thus, identifying contributing factors that are amendable to intervention is a critical public health focus.

Depletion of essential micronutrient reserves throughout pregnancy can increase a woman’s risk for maternal depression [10,11]. Iodine is an essential micronutrient and the main element required for the synthesis of thyroid hormones, thyroxine (T4) and triiodothyronine (T3), which regulate multiple processes, including growth, metabolism, and reproduction. Thyroid hormones play important roles in modulating metabolic activity in the brain [12]. Thyroid dysfunction has also been linked to perinatal [13] and postpartum depression [13,14,15]. Iodine deficiency is common during pregnancy due not only to increased renal clearance [15] but also because increased maternal levels are needed to sustain maternal and fetal needs such that iodine requirements are 50% higher during pregnancy and lactation than any other period in the life course [16]. Thus, women of childbearing age are especially vulnerable to the adverse consequences of insufficient iodine intake, and an adequate supply of iodine nutrition before and during pregnancy is essential to adjust thyroid function to meet the increasing demands of pregnancy. Because of successful programs of universal salt iodization in formerly severely iodine-deficient regions, public health concerns have shifted from severe to mild-to-moderate iodine deficiency, which remains prevalent in many regions, especially among pregnant women [17]. Dietary guidelines set by the Institute of Medicine (IOM) recommends an estimated average requirement (EAR) of 160 µg/day, with 220 µg/day [18] being the recommended dietary allowance (RDA) during pregnancy, while the World Health Organization recommends a nutrient intake of iodine of 220 µg/day. Women of reproductive age, especially during pregnancy, fall short of these optimal dietary iodine guidelines.

Elucidating the link between habitual iodine intake during pregnancy and postpartum mood disorders remains understudied, and results have been mixed. One study in a population of pregnant women with mild-to-moderate iodine deficiency found that low dietary iodine levels in the second trimester were associated with higher perinatal and 6-month postpartum depression scores, while supplemental iodine intake was linked to higher postpartum depression [19]. Although it has been suggested that an abrupt increase in iodine from supplements, particularly in women with low intake from food, could cause a “stunning effect” on the thyroid gland and a temporary imbalance in thyroid hormones [20] that might affect mood, Wang et al., 2020 [21] found no differences in depression score 1-month postpartum and no difference in thyroid stimulating hormone (TSH). However, they reported a difference in free thyroxine (FT4) between groups that received iodine supplements (150 µg/day—the recommended supplemental intake level by the American Thyroid Association), as compared to those who received supplements without iodine and those who did not receive supplements. Depression scores were higher in the iodine supplement group, but the difference was not statistically significant [21]. Furthermore, no prior study has considered anhedonia symptoms separate from depressive symptom scores in the peripartum period with respect to mild-to-moderate iodine deficiency in pregnant women.

This study conducted in an ethnically diverse pregnancy cohort in the Northeastern United States (U.S.) examined associations between prenatal habitual dietary iodine intake alone and in combination with its supplemental counterpart and maternal depressive and anhedonia symptoms assessed in pregnancy and 6 months postpartum.

## 2. Materials and Methods

### 2.1. Study Population

Participants were mothers enrolled in the PRogramming of Intergenerational Stress Mechanisms (PRISM) study, an ongoing prospective pregnancy cohort originally designed to examine the influence of perinatal stress, maternal nutrition, and other environmental exposures on child development and health. Pregnant women were recruited between March 2011 and April 2020 from prenatal clinics in Boston and New York City hospitals. Eligibility included English or Spanish speaking, ≥18 years at enrollment, and carrying a singleton pregnancy. Women were excluded if they reported drinking ≥7 alcoholic beverages/week before pregnancy recognition or any alcohol consumption after becoming pregnant and having an infant born with congenital anomalies or neurological dysfunction that would impede their ability to participate in longitudinal follow-up. PRISM was conducted in accordance with prevailing ethical principles, and all procedures were approved by the human subject committees at the Brigham and Women’s Hospital and Icahn School of Medicine at Mount Sinai; written informed consent was obtained in the participants’ primary language. From a total of 1731 eligible mothers, after excluding mothers with more than one child from this study, 837 mothers with non-missing prenatal dietary iodine information were included in these analyses.

### 2.2. Exposures

Maternal Iodine: Prenatal dietary intake in the past 3 months was assessed in the second trimester of pregnancy with an interviewer-administered modified BLOCK98 FFQ (Block 2006_Bodnar FFQ, version 98.2, NutritionQuest, Berkeley, CA, USA) consisting of 120 food and beverage items [22,23]. The BLOCK98 FFQ incorporates dietary and questionnaire changes suggested by American national consumption data collected from the third National Health and Nutrition Examination Survey (NHANES III) [24,25] and has been validated in multi-cultural populations, including pregnant women [23,26]. The FFQ was administered in English or Spanish by bilingual research staff, reviewed for completion, and processed through the online BLOCK Dietary Data Systems (Berkeley, CA, USA) for micronutrient analysis using software developed at the National Cancer Institute (NCI). For each item on the FFQ, an average daily nutrient intake was calculated based on the nutrient content of the item and the frequency and portion size consumed. Nutrient values were calculated by multiplying the nutrient content of the food or beverage by the gram weight and frequency and summing across all food items. To compute iodine micronutrient values in foods, beverages, and dietary supplements, we used the most recent USDA, FDA, and ODS-NIH Database for the Iodine Content of Common Foods [27,28,29] and other scientific resources [30,31,32]. Supplementary data were linked to the NHANES dietary supplement files. For specific iodine-containing supplement use analysis, we identified supplements by their inclusion of iodine based on their ingredient identification codes on the dietary supplement database file. Estimates of iodine intake from dietary sources were generated through linkage to dietary intake tools and to the data generated by the NHANES. To estimate updated iodine data from reported foods, the database developed by USDA version 3.0 [27], the Food and Drug Administration (FDA) Center for Food Safety and Applied Nutrition in College Park, MD, and the Office of Dietary Supplements, NIH, were utilized, along with the Office of Dietary Supplements, National Institutes of Health. After matching the consumed dietary supplement product with the ingredient information from the NHANES database, we categorized study participants as users or nonusers of dietary supplements with iodine. For these analyses, iodine intake from food and from food and supplements combined were categorized as <100 µg/day, 100–<160 µg/day, 160–220 µg/day (reference), 221–<400 µg/day, and ≥400 µg/day (due to the few mothers at or exceeding this intake level (12% and 15%, respectively, for dietary iodine and combined dietary and supplemental iodine). The reference group was chosen to reflect the IOM EAR of 160 µg/day and the RDA of 220 µg/day during pregnancy [18].

### 2.3. Outcomes

Global EPDS Score: The 10-item Edinburgh Postnatal Depression Scale (EPDS) can be used to assess depressive symptoms in the past 7 days as a unidimensional scale and as multidimensional subscales, including depressive and anhedonia symptoms in pregnant, postnatal, and non-pregnant women and it is validated in both English and Spanish populations [33,34,35]. The 10-item EPDS [8] was used to assess global antepartum depression in the second trimester, and postpartum depression was assessed 6 months after delivery. We focused on 6-month PPD in this study because it represents stable depression episodes unrelated to pregnancy. Women completed the EPDS 6-month postpartum either by telephone or during an in-person laboratory visit, and some completed IT at both visits (*n* = 455 (54%)). We used as the postpartum score non-missing values from either the telephone or the laboratory visits, and when both were available, we chose the higher total score of the two. Items included: “1: able to laugh”, “2: looking forward”, “3: self-blaming”, “4: worrying”, “5: scared”, “6: things get on top of me (overwhelmed)”, “7: difficult to sleep”, “8: feeling sad”, “9: crying”, and “10: the thought of self-harming”. Items were scored on a Likert scale from 0, indicating the most favorable condition, to 3, indicating the least favorable condition for each item. The range of the global EPDS score in this sample was 0–29 in the antepartum and postpartum periods.

The global EPDS score (antepartum and 6 months postpartum) was categorized into ≥10 vs. <10, a threshold that has been used as a consensus cutoff for screening for a concern of clinical depression [36]. A score of ≥10 on the EPDS scale has a reported sensitivity and specificity of 0.85 (95% CI: 0.79 to 0.90) and 0.84 (95% CI: 0.79 to 0.88), respectively [36]. We also considered a cutoff score of ≥13 vs. <13 (which is also commonly used) in a sensitivity analysis. Existing literature suggests that the EPDS can be used to assess multidimensional perinatal psychological functioning, although originally developed as a unidimensional scale. An exploratory and confirmatory factor analysis in the PRISM cohort identified 2-factor subconstructs (anhedonia and depressive symptoms) from the global EPDS [35]. The anhedonia subconstruct loaded on items 1 and 2, while the depression subconstruct loaded on items 3–9. Item 10 was not considered in the subconstruct analysis due to a very rare positive endorsement rate (only 0.8%).

Subscale Scores: We considered a depressive symptom subscale score using 7 items (questions 3–9) scored on the same 0 to 3 Likert scale; the range in our sample was 0–21. For data analysis, we used a median split of the total sub-score value (≥4 vs. <4) to indicate higher depressive vs. lower depressive subscale symptoms, respectively, for both the prenatal and 6-month postpartum periods.

Item 1: “In the past 7 days, I have been able to laugh and see the funny side of things”, and item 2: “In the past 7 days, I have looked forward with enjoyment to things” assessed anhedonia subscale symptoms. Responses were scored on the same 0 to 3 Likert scale; the range in our sample was 0 to 6. We categorized those with a total score of 0 as having no anhedonia symptoms and those with a total score ≥ 1 as having symptoms of anhedonia, both during pregnancy and 6 months postpartum.

Covariates: We considered as adjustment covariates child sex (male, female); maternal age at birth (in years), parity (nulliparous, primiparous, or multiparous); self-reported pre-pregnancy depression diagnosis (yes or no); educational attainment (high school or less, and more than high school); race/ethnicity (Black/Hispanic Black, non-Black Hispanic, non-Hispanic white, other) and previous treatment for a thyroid disorder based on response to the self-reported question on treatment for thyroid disease in the past and abstraction from medical records.

### 2.4. Data Analysis

Descriptive statistics of frequencies and percentages summarized demographic variables, while the median (IQR) summarized dietary and supplemental iodine intake levels for the different demographic variables. Differences in iodine intake levels were assessed using the Wilcoxon rank-sum test. Missing data were as low as 2.4% for maternal race/ethnicity and as high as 31.5% for postpartum depression. Missing data were imputed using chained equations [37], with a total of 40 datasets imputed using 500 between imputation iterations. Regression analyses were conducted on both the imputed and unimputed datasets, and the results were qualitatively similar; therefore, imputed regression results are presented, and unimputed results are available for review in the supplemental material. Logistic regression estimating odds ratios and 95% confidence intervals (CIs) estimated associations separately between dietary and combined dietary and supplementary iodine intake levels in the 2nd trimester of pregnancy separately with antepartum and 6-month postpartum depressive and anhedonia symptoms. We evaluated effect measure modification by child sex and maternal race/ethnicity, setting a *p*-value for significant modification to *p* < 0.1, and the results indicated that both factors were not significant effect modifiers and were thus adjusted for as confounders in the main analyses. Due to running multiple models on correlated scales, we adjusted for multiple comparisons at a Bonferroni correction *p*-value < 0.025, considered statistically significant.

## 3. Results

### 3.1. Characteristics of Study Participants and Prenatal Iodine Intake

Most women were Black/Hispanic Black (43%) and non-Black Hispanics (35%). More than one-third reported having a high school education or less, and 25% reported a diagnosis of depression pre-pregnancy. The overall median (IQR) dietary and supplemental iodine intake level was 187 (116, 315) µg/day. We observed significant differences in dietary and supplemental iodine intake levels according to maternal race/ethnicity (*p* = 0.05), pre-pregnancy depression (*p* = 0.02), and prenatal global EPDS ≥ 10 (*p* = 0.05). The median (interquartile range, IQR) dietary and supplemental iodine intake among Black/Hispanic Black (198 (115, 337) µg/day) and non-Black Hispanic women (195 (126, 323) µg/day) was higher than the overall median intake level of 187 (116, 315) µg/day and was also higher than the median intake of dietary and supplemental iodine than the other racial/ethnic groups. Those reporting a diagnosis of depression pre-pregnancy had statistically significant (*p* = 0.02) higher median intake levels of dietary and supplemental iodine at 204 (131, 378) µg/day than those without pre-pregnancy depression whose median intake level was 181 (112, 304) µg/day. Similarly, those with a global EPDS ≥ 10 during pregnancy had a statistically significant (*p* = 0.05) higher median intake of 203 (127, 345) µg/day than those with a prenatal EPDS < 10 with a median intake of 177 (112, 312) µg/day. On the contrary, women with a 6-month postpartum global depressive symptom score EPDS ≥ 10 had lower but non-significant (*p* = 0.6) median dietary and supplemental iodine intake levels when compared to women with a postnatal global EPDS < 10 (168 (116, 324) vs. 191 (117, 319) µg/day, respectively). Likewise, women with postpartum anhedonia symptoms had significantly (*p* = 0.04) lower dietary/supplemental iodine intake levels 158 (108, 321) µg/day than those without postpartum anhedonia symptoms, 198 (126, 319) µg/day. There were no differences in iodine intake during pregnancy and the postpartum period for the depressive symptom subscale score (Table 1).

### 3.2. Regression Results

**Figure 1:**
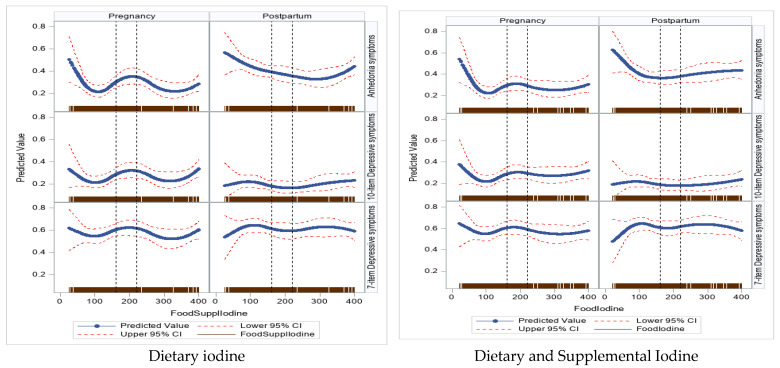
Univariate dose–response curves for prenatal iodine intake with pregnancy and postpartum mental health outcomes.

Figure 1 shows univariate dose–response curves for iodine intake (dietary alone (panel 1) and dietary/supplemental iodine (panel 2)) with antepartum and postpartum depressive (10-item global and 7-item subscale scores) and anhedonia symptoms. The shape of the dose–response for antepartum mood disorder symptoms was different from the shape of the dose–response observed in the postpartum period. Based on these plots, iodine intake, whether below or above recommended levels in pregnant women, was associated with greater global depressive and anhedonia symptoms, particularly in the postpartum period.

#### 3.2.1. Iodine Intake and Global EPDS Score—Antepartum

There was a suggested association between dietary iodine intake and greater odds of antepartum depression (EPDS ≥ 10) for intake levels greater than the recommended thresholds in the unadjusted and adjusted models. When compared to the reference (IOM recommended) of 160–220 µg/day, those in the 201–<400 µg/day and ≥400 µg/day had higher adjusted odds of having a global EPDS ≥ 10, OR = 1.09 (95% CI: 0.65, 1.83) and OR = 1.16 (95% CI: 0.65, 2.08), respectively. The estimates were similar for the combined dietary and supplemental iodine (Table 2).

#### 3.2.2. Postpartum

There was a suggested association between low and high levels (relative to the reference of 160–220 µg/day) of the combined dietary and supplemental iodine intake and greater unadjusted and adjusted odds of the global 6-month postpartum EPDS score ≥ 10. Specifically, women with intake levels classified as <100 µg/day, 100–<160 µg/day, >220–<400 µg/day and ≥400 µg/day had greater adjusted odds of global postpartum depressive symptoms score ≥ 10 than the reference intake group (aOR = 1.39 (95% CI: 0.74, 2.63), 1.44 (95% CI: 0.80, 2.59), 1.31 (95% CI: 0.73, 2.35), and 1.12 (95% CI: 0.58, 2.17), respectively). We found a similar trend for dietary iodine alone but with smaller magnitudes (Table 3).

#### 3.2.3. Iodine Intake and Anhedonia and Depressive Subscales Symptom Scores—Antepartum

Dietary iodine intake appears to be associated with greater odds of antepartum anhedonia for intake levels lower and greater than the recommended thresholds in unadjusted and adjusted models. When compared to the reference of 160–220 µg/day, those with intake of 100–<160 µg/day had higher adjusted odds of antepartum anhedonia symptoms, OR = 1.17 (95% CI: 0.72, 1.90). Likewise, those with the highest dietary intake levels ≥ 400 µg/day had higher adjusted odds of antepartum anhedonia of OR = 1.29 (95% CI: 0.72, 2.30). These estimates for low and high prenatal iodine intake levels are suggestive of associations in the detrimental direction to maternal mental health, although not statistically significant at the *p* < 0.05 threshold, likely due to the modest sample size. The estimates were similar for the combined dietary and supplemental iodine but with slightly attenuated estimates (Table 2). We found no meaningful associations between dietary and dietary/supplemental iodine intake with the 7-item depressive symptom subscale score during pregnancy (Supplementary Table S4).

#### 3.2.4. Postpartum

Low and high dietary iodine intakes (relative to the reference of 160–220 µg/day) were associated with greater unadjusted and adjusted odds of 6-month postpartum anhedonia symptoms. Specifically, women with intake levels classified as <100 µg/day, 100–<160 µg/day, >220–<400 µg/day, and ≥400 µg/day had higher adjusted odds of 6-month postpartum anhedonia of 1.74 (95% CI: 1.08, 2.79), 1.25 (95% CI: 0.80, 1.99), 1.31 (95% CI: 0.82, 2.10), and 1.47 (95% CI: 0.86, 2.51), respectively, as compared to women in the reference intake level. We found similar trends for dietary and supplemental iodine but with slightly smaller magnitudes (Table 3). We found no meaningful associations between dietary and dietary/supplemental iodine intake with the 7-item depressive symptom subscale score 6 months postpartum (Supplementary Table S4).

Results of complete case analysis were similar in magnitude and direction for both the prenatal, *n* = 705 (Supplementary Table S1) and the postpartum period, *n* = 544 (Supplementary Table S2). Results of the global depressive symptom score with a cutoff score of ≥13 are presented in Supplementary Table S3.

## 4. Discussion

Our findings suggest an association (U-shaped) of dietary/supplemental iodine intake both below and above the IOM recommended intake thresholds for pregnant women with worse mental health outcomes in the postpartum period. For both the global depressive and anhedonia subscale symptoms, our findings were more compelling in the postpartum period than during pregnancy. Indeed, our findings suggest that a higher intake level might be needed during pregnancy to accommodate the needs of the mother and developing fetus while intake in the recommended IOM range, the EAR (160 µg/day) and RDA (220 µg/day) appear sufficient to mitigate poorer maternal health outcomes after birth, specifically depressive and anhedonia symptoms 6 months postpartum. Notably, our estimates of PPD (based on an EPDS ≥ 10) of 20% is similar to reports of 17–20% [1,38] previously reported among pregnant women.

Abnormalities in thyroid function are more prevalent after delivery, with up to 7% of all new mothers experiencing thyroid dysfunction postpartum, compared with a prevalence of 3–4% in the general population [39]. Mood disorders are common in individuals with thyroid conditions (hyper or hyperthyroidism) and autoimmune thyroiditis. Thyroid autoimmunity during pregnancy and in the weeks after childbirth is associated with an increased risk of developing PPD [13,14,40]. Iodine is important for thyroid hormone secretion and the proper functioning of the thyroid gland [41]. Although most patients with depression do not have overt thyroid disease, subclinical hypothyroidism is found in 15% to 20%, and it is the most common thyroid dysfunction in patients with mood disorders [41]. Thyroid dysfunction may thus be a mechanism by which this association occurs, and an explanation for the modest effect sizes may be an indication of iodine intake levels being a distal/upstream risk factor as opposed to more proximal thyroid dysfunction indicators such as high levels of hormones like TSH reported to be associated with worse maternal mental health in the postpartum period.

Studies have found that appropriate iodine supplementation during pregnancy can reduce thyroid volume, decrease serum thyroglobulin level, inhibit the increase in serum TSH level, and reduce the risk of low FT4 levels [20], precursors for poor maternal mental health outcomes. Unlike some prior studies, we found little discrepancy in maternal mental health outcomes for dietary iodine alone when compared to dietary and supplemental iodine at the recommended intake levels, albeit our findings were not always consistent between these two iodine intake pathways. The non-consistent results for iodine from food and iodine from food and supplements in our study may be explained by differences in the effect of long-term habitual iodine intake from food as compared to supplemental iodine. The impact of iodine from supplements depends on habitual iodine intake from food, timing of introduction, dose, frequency, and duration of use [42], which we were unable to consider fully in our data analysis. In mild-to-moderate iodine deficiency, the body adapts to low iodine intake, for example, by increasing the size of the thyroid gland. When iodine intake is increased by supplements or by salt iodization, it may take months to adapt to the new intake level, which can lead to a temporary inhibition of thyroid hormone production [43]. Duration of supplement use was not asked of PRISM cohort participants, but if supplements were used intermittently or for a short duration, it is possible that mothers may not have fully adapted to new intake levels. Another likely explanation for our findings is reverse causation, given that women with underlying psychopathology may be prone to using multivitamin supplements that can abruptly increase iodine levels and, as a side effect trigger, thyroid function imbalances known to affect mood [20]. Differences in the direction of effects for antepartum and postpartum mood states may also be due to physiological differences between pregnancy and the postpartum period.

The results herein point to the importance of considering depressive and anhedonia symptoms in pregnant women and women in the postpartum period, especially given supplement use drops sharply during the postpartum period. Indeed, our findings appear to be driven more by anhedonia. Given that anhedonia questions are a subset of the 10-item EPDS depression scale, the depressive symptom results may have also been driven by anhedonia. When we analyzed the data looking at depressive symptoms without considering the anhedonia questions, the associations were less compelling (Supplementary Table S4).

### Strengths and Limitations

We acknowledge both the strengths and potential weaknesses of this study. These analyses are the first to examine these associations in a multi-ethnic, socioeconomically diverse U.S. sample, adding to the literature supporting an association between iodine intake in pregnancy and mood disorders in the antepartum and postpartum periods. We further add to the prior literature by considering both depressive and anhedonia symptoms assessed using the validated and widely used EPDS. Prenatal iodine intake was measured with a widely used and validated FFQ for assessing average habitual dietary intake in pregnant women, and we incorporated intake from both dietary sources and supplements. Nonetheless, the use of FFQs may result in underreporting of iodine intake. Future work should consider adding 24 h dietary recalls or food records as well as biomarkers of bioavailable iodine such as repeated urinary iodine concentrations [44,45] or cumulative prenatal iodine stores assessed with placenta [46] in relation to mood disorders in pregnancy and the postpartum period, particularly when evaluating intake above/below the suggested thresholds [47]. While we used a valid approach to characterizing anhedonia symptoms, scales developed specifically to assess anhedonia with an increased number of items (e.g., Specific Loss of Interest and Pleasure Scale (SLIPS) [48] and Temporal Experience of Pleasure Scale (TEPS) [49]) may provide greater precision in the assessment of this domain. However, it is not uncommon for symptoms of anhedonia to be assessed with a small number of items on a depression symptom scale. For instance, a single question on the 17-item Hamilton Depression Rating Scale, 4 items on the 21-item Beck Depression Inventory, and 4 items on the 30-item Inventory of Depressive Symptoms [50]. Higher scores on the EPDS scale provide an indication for the presence of depressive symptoms rather than the severity of depression or DSM-V depression diagnosis. Severity may be an important construct to study together with the anhedonia subtype, given evidence that the presence of anhedonia has been associated with increased depressive symptom severity and is a negative predictor of treatment response [2]. Recognized predisposing factors for depression include genetic susceptibility, previous psychiatric illnesses, adverse life events, marital disharmony, lack of a confiding relationship, housing problems, or other socioeconomic problems [51]. While we adjusted for many of these predisposing factors, others could not be considered as data were not available (e.g., genetics and other social conditions), thus leaving the possibility for residual confounding. These results warrant replication in other diverse populations with larger sample size cohorts to corroborate the growing evidence linking prenatal iodine intake and mood disorders in pregnancy and postpartum women, especially since iodine intake is modifiable and can be intervened upon. Larger sample size studies would also allow one to consider potential effect modifiers of these associations (e.g., race/ethnicity and socioeconomic status) and modification by multiple essential trace elements, notably, those that have been found to play pivotal roles in regulating neurodevelopment and mood depressive disorders, Se, Zn, Cu, and Mo (alongside iodine) [52].

## 5. Conclusions

Dietary/supplemental iodine intake lower and higher than the recommended threshold for pregnant women appears to adversely affect the mental health of women in this ethnically and socioeconomically diverse sample, particularly in the postpartum period, while intake levels higher than the recommended threshold appear beneficial to maternal mental health during pregnancy.

These results warrant future studies in other diverse populations with larger sample sizes to corroborate the growing evidence linking prenatal iodine intake and mood disorders in pregnant and postpartum women, especially since low iodine levels appear to be a risk factor amenable to behavioral, clinical, biomedical, and public health interventions.

## Figures and Tables

**Table 1:** Distribution of socio-demographic characteristics according to dietary and supplemental iodine intake, PRISM (Programming of Intergenerational Stress Mechanisms) pregnancy cohort.

	N	Percent	Food and Supplement Iodine (µg/Day), Median (IQR) ^#^	*p*-Value
Overall	837	100	187 (116, 315)	
Maternal factors				
Age at birth, years, mean (SD) *	29.8 (5.92)			
Age at birth, years				0.4
18–<25	194	23.2	175 (111, 324)	
25–<35	473	56.5	188 (114, 310)	
≥35	170	20.3	198 (127, 320)	
Race/ethnicity				0.05
Non-Hispanic White	142	17.3	151 (110, 269)	
Black/Hispanic Black	352	43	198 (115, 337)	
Non-Black Hispanic	288	35.1	195 (126, 323)	
Other	37	4.52	153 (107, 281)	
Missing	18			
Education				0.6
High school or less	321	39.2	185 (111, 331)	
More than high school	496	60.7	191 (118, 311)	
Missing	20			
Depression pre-pregnancy				0.02
Yes	208	25.3	204 (131, 378)	
No	614	74.7	181 (112, 304)	
Missing	15			
Pregnancy depressive symptoms (Global EPDS)				0.05
<10	538	71.5	177 (112, 312)	
≥10	214	28.5	203 (127, 345)	
Missing	85			
Postpartum depressive symptoms (Global EPDS)				0.6
<10	454	79.2	191 (117, 319)	
≥10	119	20.8	168 (116, 324)	
Missing	264			
Pregnancy anhedonia symptoms				0.6
0	552	73.5	185 (115, 324)	
1–6	199	26.5	193 (120, 317)	
Missing	86			
Postpartum anhedonia symptoms				0.04
0	391	68.2	198 (126, 319)	
1–6	182	31.8	158 (108, 321)	
Missing	264			
Pregnancy depressive subscale symptoms				0.5
<4	339	45.1	180 (111, 318)	
≥4	413	54.9	193 (118, 322)	
Missing	85			
Postpartum depressive subscale symptoms				0.8
<4	292	51.0	188 (117, 323)	
≥4	281	49.0	187 (118, 315)	
Missing	264			
Thyroid disease ^§^				0.4
Yes	52	6.33	170 (113, 278)	
No	769	93.7	190 (116, 320)	
Missing	16			
Child sex				0.8
Female	422	50.5	181 (116, 323)	
Male	413	49.5	192 (115, 305)	
Missing	2			

* SD—standard deviation; ^#^ IQR—interquartile range. ^§^ Treatment for a thyroid disease was based on self-report and medical record extraction. *p*-value for Wilcoxon–Mann–Whitney rank-sum test.

**Table 2:** Prenatal Iodine intake and maternal pregnancy mental health symptoms scores.

	Prenatal Anhedonia Symptoms		Prenatal Global EPDS Score	
	Odds Ratio (95% CI)		Odds Ratio (95% CI)	
Dietary Iodine Intake	Crude	Adjusted	Crude	Adjusted
<100 µg/day	1.03 (0.61, 1.73)	0.99 (0.58, 1.67)	0.87 (0.52, 1.47)	0.82 (0.48, 1.40)
100–<160 µg/day	1.14 (0.71, 1.84)	1.17 (0.72, 1.90)	0.97 (0.60, 1.57)	0.96 (0.59, 1.57)
160–220 µg/day	Ref.	Ref.	Ref.	Ref.
>220–<400 µg/day	1.05 (0.63, 1.75)	1.04 (0.62, 1.74)	1.13 (0.68, 1.86)	1.09 (0.65, 1.83)
≥400 µg/day	1.40 (0.80, 2.47)	1.29 (0.72, 2.30)	1.35 (0.77, 2.36)	1.16 (0.65, 2.08)
Dietary and supplemental iodine				
<100 µg/day	0.98 (0.58, 1.67)	0.96 (0.56, 1.65)	0.89 (0.52, 1.53)	0.86 (0.49, 1.50)
100–<160 µg/day	1.00 (0.62, 1.65)	1.04 (0.63, 1.71)	0.93 (0.56, 1.53)	0.93 (0.56, 1.56)
160–220 µg/day	Ref.	Ref.	Ref.	Ref.
>220–<400 µg/day	0.97 (0.60, 1.58)	0.97 (0.59, 1.60)	1.08 (0.66, 1.76)	1.07 (0.65, 1.77)
≥400 µg/day	1.19 (0.69, 2.05)	1.12 (0.64, 1.94)	1.33 (0.78, 2.29)	1.18 (0.68, 2.06)

Models were adjusted for child sex, maternal age at birth, maternal race/ethnicity, education, pre-pregnancy depression, parity, and previous treatment for a thyroid disease. Anhedonia was based on items 1 and 2 of the 10-item EPDS (Edinburg depression scale), and those with a score of ≥1 were categorized with anhedonia symptoms and 0 with no anhedonia symptoms. Global depressive symptoms were based on a score ≥ 10 on the 10-item EPDS depression scale. Reference for iodine intake was based on the IOM, EAR (160 µg/day), and RDA (220 µg/day) for pregnant women in the US.

**Table 3:** Prenatal iodine intake and maternal 6-month postpartum mental health outcomes symptoms scores.

	Postpartum Anhedonia Symptoms		Postpartum Global EPDS Score	
	Odds Ratio (95% CI)		Odds Ratio (95% CI)	
Dietary Iodine Intake	Crude	Adjusted	Crude	Adjusted
<100 µg/day	1.74 (1.09, 2.78)	1.74 (1.08, 2.79)	1.20 (0.68, 2.13)	1.20 (0.66, 2.19)
100–<160 µg/day	1.23 (0.79, 1.90)	1.25 (0.80, 1.99)	1.15 (0.67, 1.98)	1.17 (0.67, 2.05)
160–220 µg/day	Ref.	Ref.	Ref.	Ref.
>220–<400 µg/day	1.32 (0.83, 2.10)	1.31 (0.82, 2.10)	1.16 (0.66, 2.06)	1.12 (0.62, 2.01)
≥400 µg/day	1.53 (0.90, 2.60)	1.47 (0.86, 2.51)	1.21 (0.63, 2.30)	1.03 (0.53, 2.02)
Dietary and supplemental iodine intake				
<100 µg/day	1.72 (1.06, 2.79)	1.74 (1.07, 2.83)	1.35 (0.73 (2.49)	1.39 (0.74, 2.63)
100–<160 µg/day	1.23 (0.78, 1.94)	1.26 (0.80, 1.99)	1.38 (0.78, 2.45)	1.44 (0.80, 2.59)
160–220 µg/day	Ref.	Ref.	Ref.	Ref.
>220–<400 µg/day	0.94 (0.60, 1.48)	0.93 (0.59, 1.47)	1.35 (0.77, 2.39)	1.31 (0.73, 2.35)
≥400 µg/day	1.29 (0.78, 2.14)	1.22 (0.73, 2.04)	1.32 (0.70, 2.50)	1.12 (0.58, 2.17)

Models were adjusted for child sex, maternal age at birth, maternal race/ethnicity, education, pre-pregnancy depression, parity, and previous treatment for a thyroid disease. Anhedonia was based on items 1 and 2 of the 10-item EPDS (Edinburg depression scale), and those with a score of ≥1 were categorized with anhedonia symptoms and 0 with no anhedonia symptoms. Global depressive symptoms were based on a score ≥ 10 on the 10-item EPDS depression scale. Reference for iodine intake was based on the IOM, EAR (160 µg/day), and RDA (220 µg/day) for pregnant women in the US. Bold estimates indicate statistical significance at a Bonferroni corrected *p*-value < 0.025.

## Data Availability

The original contributions presented in the study are included in the article/Supplementary Material, further inquiries can be directed to the corresponding author.

## Supplementary Materials

The following supporting information can be downloaded at: https://www.mdpi.com/article/10.3390/nu16111771/s1, Table S1: Prenatal Iodine intake and maternal pregnancy mental health symptoms scores; Table S2: Prenatal Iodine intake and maternal postpartum mental health symptoms scores; Table S3: Prenatal iodine intake and maternal pregnancy and 6-months postpartum global depression score; Table S4: Prenatal iodine intake and maternal pregnancy and 6-months postpartum depressive subscale scores; Table S5: Covariate balance between those participants included and excluded from current analyses.
