# Supplementary material for: Prenatal Iodine Intake and Maternal Pregnancy and Postpartum Depressive and Anhedonia Symptoms: Findings from a Multiethnic US Cohort
Source: Nutrients. 2024 Jun 5;16(11):1771. doi: 10.3390/nu16111771 (PMC11174687; doi:10.3390/nu16111771)
Supplement: Supplementary file 1 [file nutrients-16-01771-s001.zip › nutrients-3025964-supplementary.pdf]

SUPPLEMENTARY TABLES

COMPLETE CASE ANALYSIS

Supplementary Table S1. Prenatal Iodine intake and maternal pregnancy mental health symptoms scores

	Prenatal Anhedonia symptoms		Prenatal Global EPDS score	
	Odds Ratio (95% CI)		Odds Ratio (95% CI)	
	**Crude**	**Adjusted**	**Crude**	**Adjusted**
**Dietary Iodine**	n=751	n=705	n=752	n=706
<100μg/day	0.99 (0.56, 1.73)	0.89 (0.49, 1.62)	0.79 (0.45, 1.36)	0.88 (0.49, 1.59)
100-<160μg/day	1.15 (0.69, 1.93)	1.04 (0.60, 1.78)	0.90 (0.55, 1.48)	0.95 (0.55, 1.62)
160-220μg/day	Ref.	Ref.	Ref.	Ref.
>220-<400 μg/day	1.01 (0.58, 1.77)	0.99 (0.55, 1.77)	1.03 (0.61, 1.75)	1.09 (0.61, 1.92)
≥400 μg/day	1.40 (0.77, 2.57)	1.35 (0.72, 2.53)	1.29 (0.72, 2.30)	1.20 (0.64, 2.25)
**Dietary and supplemental iodine**				
<100μg/day	0.89 (0.50, 1.58)	0.79 (0.43, 1.45)	0.78 (0.44, 1.39)	0.89 (0.48, 1.65)
100-<160μg/day	0.96 (0.56, 1.62)	0.81 (0.47, 1.41)	0.90 (0.53, 1.51)	0.93 (0.53, 1.62)
160-220μg/day	Ref.	Ref.	Ref.	Ref.
>220-<400 μg/day	0.93 (0.55, 1.57)	0.88 (0.51, 1.51)	1.03 (0.62, 1.72)	1.12 (0.65, 1.94)
≥400 μg/day	1.15 (0.65, 2.05)	1.08 (0.59, 1.97)	1.28 (0.73, 2.25)	1.16 (0.63, 2.13)

Models were adjusted for maternal age at birth, child sex, maternal race/ethnicity, education, pre-pregnancy depression, parity, previous treatment for a thyroid disease. Anhedonia was based on items 1 and 2 of the 10-items EPDS (Edinburg depression scale) and those with score of ≥1 were categorized with anhedonia symptoms and 0 with no anhedonia symptoms. Global depressive symptoms was based on score ≥10 on the 10-item EPDS depression scale. Reference for iodine intake was based on the IOM, EAR (160 μg/day) and RDA (220 μg/day) for pregnant women in the US.

Dietary Iodine ≥400 μg/day (relative to 160-220μg/day) was associated with increased unadjusted and adjusted odds of prenatal anhedonia symptoms, 1.40 (0.77, 2.57) and 1.35 (0.72, 2.53) respectively. We also found a higher unadjusted and adjusted odds of depressive symptoms during pregnancy for dietary iodine intake levels ≥400 μg/day relative to the reference level of 160-220μg/day. The corresponding OR (95% CI) were 1.29 (0.72, 2.30) and 1.20 (0.64, 2.25) respectively (Supplementary Table 1).

Supplementary Table S2. Prenatal Iodine intake and maternal postpartum mental health symptoms scores

	Postpartum anhedonia symptoms		Postpartum Global EPDS score	
	Odds Ratio (95% CI)		Odds Ratio (95% CI)	
	**Crude**	**Adjusted**	**Crude**	**Adjusted**
**Dietary Iodine**	n=573	n=544	n=573	n=544
<100μg/day	1.80 (0.99, 3.25)	1.70 (0.91, 3.18)	0.93 (0.47, 1.84)	0.98 (0.47, 2.05)
100-<160μg/day	1.08 (0.62, 1.89)	1.04 (0.58, 1.87)	1.04 (0.56, 1.92)	1.04 (0.53, 2.03)
160-220μg/day	Ref.	Ref.	Ref.	Ref.
>220-<400 μg/day	0.99 (0.53, 1.85)	0.99 (0.52, 1.87)	0.76 (0.37, 1.54)	0.73 (0.34, 1.54)
≥400 μg/day	1.23 (0.63, 2.39)	1.14 (0.57, 2.29)	1.12 (0.54, 2.34)	0.93 (0.42, 2.06)
**Dietary and supplemental Iodine**				
<100μg/day	1.79 (0.96, 3.32)	1.87 (0.97, 3.58)	0.92 (0.44, 1.93)	1.20 (0.54, 2.67)
100-<160μg/day	1.21 (0.68, 2.15)	1.22 (0.66, 2.24)	1.38 (0.72, 2.63)	1.58 (0.78, 3.22)
160-220μg/day	Ref.	Ref.	Ref.	Ref.
>220-<400 μg/day	0.78 (0.43, 1.42)	0.82 (0.44, 1.54)	0.78 (0.39, 1.55)	0.86 (0.41, 1.81)
≥400 μg/day	1.09 (0.57, 2.06)	1.07 (0.54, 2.10)	1.17 (0.57, 2.40)	1.09 (0.49, 2.40)

Models were adjusted for maternal age at birth, child sex, maternal race/ethnicity, education, pre-pregnancy depression, parity, previous treatment for a thyroid disease. Anhedonia was based on items 1 and 2 of the 10-items EPDS (Edinburg depression scale) and those with score of ≥1 were categorized with anhedonia symptoms and 0 with no anhedonia symptoms. Global depressive symptoms was based on score ≥10 on the 10-item EPDS depression scale. Reference for iodine intake was based on the IOM, EAR (160 μg/day) and RDA (220 μg/day) for pregnant women in the US.

In the postpartum period, high (≥400 μg/day) and low (<100μg/day) levels of dietary iodine intake were associated with greater unadjusted and adjusted odds of postpartum anhedonia. Relative to the reference dietary iodine intake of 160-220μg/day, the corresponding OR (95% CI) were respectively 1.80 (0.99, 3.25) and 1.70 (0.91, 3.18) for dietary iodine intake <100μg/day and 1.23 (0.63, 2.39) and 1.14 (0.57, 2.29) for dietary iodine intake ≥400 μg/day (Supplementary Table 2).

Supplementary Table S3. Prenatal iodine intake and maternal pregnancy and 6-months postpartum global depression score

	Pregnancy Global EPDS score		Postpartum Global EPDS score	
	Odds Ratio (95% CI)		Odds Ratio (95% CI)	
	Crude	Adjusted	Crude	Adjusted
**Dietary Iodine**				
<100μg/day	1.02 (0.51, 2.03)	0.93 (0.46, 1.90)	0.75 (0.34, 1.68)	0.75 (0.33, 1.72)
100-<160μg/day	1.26 (0.67, 2.36)	1.21 (0.63, 2.31)	1.20 (0.60, 2.40)	1.22 (0.60, 2.50)
160-220μg/day	Ref.	Ref.	Ref.	Ref.
>220-<400 μg/day	1.17 (0.60, 2.29)	1.09 (0.54, 2.17)	0.83 (0.38, 1.81)	0.78 (0.35, 1.74)
≥400 μg/day	1.13 (0.52, 2.43)	0.89 (0.40, 1.96)	1.31 (9.58, 2.96)	1.10 (0.47, 2.57)
**Dietary and supplemental Iodine**				
<100μg/day	1.35 (0.65, 2.77)	1.31 (0.63, 2.74)	0.70 (0.29, 1.68)	0.71 (0.29, 1.74)
100-<160μg/day	1.46 (0.74, 2.85)	1.46 (0.73, 2.89)	1.42 (0.69, 2.91)	1.47 (0.70, 3.09)
160-220μg/day	Ref.	Ref.	Ref.	Ref.
>220-<400 μg/day	1.16 (0.59, 2.30)	1.09 (0.54, 2.19)	0.89 (0.42, 1.91)	0.84 (0.39, 1.83)
≥400 μg/day	1.36 (0.64, 2.86)	1.11 (0.51, 2.39)	1.31 (0.59, 2.91)	1.07 (0.46, 2.45)

Models were adjusted for maternal age at birth, child sex, maternal race/ethnicity, education, pre-pregnancy depression, parity, previous treatment for a thyroid disease. Depression symptoms was based on score ≥13 on the 10-item EPDS (Edinburg depression scale). Reference for iodine intake was based on the IOM, EAR (160 μg/day) and RDA (220 μg/day) for pregnant women in the US.

Supplementary Table S4. Prenatal iodine intake and maternal pregnancy and 6-months postpartum depressive subscale scores

	Pregnancy depressive symptoms subscale score		Postpartum depressive symptoms subscale score	
	Odds Ratio (95% CI)		Odds Ratio (95% CI)	
	Crude	Adjusted	Crude	Adjusted
**Dietary Iodine**				
<100μg/day	0.73 (0.46, 1.15)	0.73 (0.46, 1.17)	0.94 (0.59, 1.50)	0.94 (0.56, 1.52)
100-<160μg/day	0.86 (0.56, 1.32)	0.84 (0.54, 1.30)	0.93 (0.60, 1.44)	0.91 (0.59, 1.42)
160-220μg/day	Ref.	Ref.	Ref.	Ref.
>220-<400 μg/day	0.80 (0.50, 1.26)	0.77 (0.48, 1.24)	1.00 (0.63, 1.58)	0.99 (0.61, 1.58)
≥400 μg/day	0.83 (0.49, 1.40)	0.76 (0.44, 1.30)	0.80 (0.48, 1.36)	0.72 (0.42, 1.23)
**Dietary and supplemental Iodine**				
<100μg/day	0.75 (0.47, 1.22)	0.76 (0.47, 1.24)	0.98 (0.60, 1.60)	0.92 (0.54, 1.58)
100-<160μg/day	0.91 (0.58, 1.43)	0.89 (0.56, 1.40)	0.89 (0.57, 1.41)	0.85 (0.53, 1.36)
160-220μg/day	Ref.	Ref.	Ref.	Ref.
>220-<400 μg/day	0.82 (0.53, 1.28)	0.79 (0.50, 1.24)	0.95 (0.61, 1.49)	0.96 (0.59, 1.56)
≥400 μg/day	0.94 (0.57, 1.56)	0.85 (0.51, 1.42)	0.80 (0.48, 1.32)	0.89 (0.38, 2.10)

Models were adjusted for maternal age at birth, child sex, maternal race/ethnicity, education, pre-pregnancy depression, parity, previous treatment for a thyroid disease. Depression symptoms was based on median split of the total score at ≥4 vs. <4 on items 3-9 of the 10-item EPDS (Edinburg depression scale). Reference for iodine intake was based on the IOM, EAR (160 μg/day) and RDA (220 μg/day) for pregnant women in the US.

Supplementary Table S5. Covariate balance between those participants included and excluded from current analyses

Overall	Excluded	Included	
	Percent	Percent	p-value
Maternal factors			
Age at birth, years, mean (SD)*	29.8 (5.9)	29.9 (5.9)	
Age at birth, years			0.1
18-<25	25	23	
25-<35	55	56	
≥35	19	21	
missing	0.52	0	
Race/ethnicity			<0.0001
NHW	4.8	17	
Black/HB	32	42	
NBH	23	34	
Other	1.7	4.6	
missing	39	2.3	
Education			<0.0001
High school or less	27	38	
More than high school	33	59	
missing	40	2.5	
Depression pre-pregnancy			<0.0001
Yes	17	25	
No	44	73	
missing	38	1.9	
Pregnancy depressive symptoms (Global EPDS)			<0.0001
<10	34	65	
≥10	15	25	
missing	51	10	
Post-partum depressive symptoms (Global EPDS)			<0.0001
<10	25	54	
≥10	736	14	
missing	67	32	
Pregnancy anhedonia symptoms			<0.0001
0	33	66	
1-6	16	24	
missing	51	10	
Post-partum anhedonia symptoms			<0.0001
0	22	47	
1-6	11	22	
missing	67	32	
Pregnancy depressive subscale symptoms			<0.0001
<4	22	40	
≥4	27	50	
missing	51	10	
Post-partum depressive subscale symptoms			<0.0001
<4	16	35	
≥4	17	33	

missing	67	32	
Thyroid disease§			<0.0001
Yes	4.5	6.2	
No	62	92	
missing	33	2	
Child sex			0.005
Female	45	50	
Male	53	49	
missing	1.6	0.3	

§Treatment for a thyroid disease was based on self-report and medical record extraction
¶p-value for chi square test
